# Mechanical behavior of deteriorated unbonded prestressed concrete hollow slabs

**DOI:** 10.1038/s41598-026-49044-7

**Published:** 2026-04-22

**Authors:** Xiaobin Li, Shuanke Zhou, Baoshan Xiang, Meng Yan, Yi Bao

**Affiliations:** 1 School of Civil Engineering, Southwest Jiaotong University, Chengdu, 610031 China; 2 Sichuan Expressway Construction and Development Group Co., Ltd., Chengdu, 610041 China; 3 Sichuan Jiaoda Engineering Detection Consulting Co., Ltd., Chengdu, 610031 Sichuan China; 4 Department of Civil, Environmental and Ocean Engineering, Stevens Institute of Technology, Hoboken, NJ 07030 USA

**Keywords:** Aging, Anchorage damage, Cracking resistance, Residual mechanical performance;, Unbonded prestressed concrete hollow slab, Engineering, Materials science

## Abstract

This study investigates the residual mechanical behavior of deteriorated unbonded prestressed concrete hollow slabs extracted from a 20-year-old highway bridge. Three full-scale slabs were tested under four-point bending to evaluate cracking resistance, load-bearing capacity, failure modes, anchorage performance, and material degradation. The exterior slab exhibited a cracking load of only 190 kN, just 12% above the design service load, indicating significantly reduced crack resistance. Failure loads ranged from 529 to 581 kN, with corresponding deflections between 287.7 and 413.6 mm. Two slabs failed prematurely due to tendon rupture at the anchorage, with ductility coefficients reduced by up to 30% compared to the ductile failure observed in Slab 1. Anchorage efficiency coefficients ranged from 0.84 to 0.88, below the code requirement of 0.95, and tendon elongation at maximum load was only 1.0–1.4%, far below the 2% minimum standard. In contrast, concrete, reinforcement, and tendons remained largely intact, with concrete compressive strength reaching 61.8 MPa, exceeding the design requirement. The residual flexural capacity decreased by 3.2–5.2% relative to theoretical values, primarily due to anchorage deterioration rather than material aging. These findings quantify the dominant role of anchorage degradation in reducing structural performance and provide critical data for assessing and rehabilitating aging unbonded PC hollow-slab bridges.

## Introduction

Unbonded prestressed concrete (PC) hollow-slab bridges are widely utilized in medium- and short-span applications^1^, due to their advantages in construction efficiency, cost-effectiveness, and standardized fabrication. However, after decades of service, many of the bridges have shown significant aging and deterioration, such as cracking, hinge joint separation, and anchorage zone damage, compromising structural integrity. Given their large population and cumulative impact of such deterioration on safety and serviceability, evaluating the residual mechanical behavior of unbonded PC hollow-slab bridges has become an urgent task. Such assessments are essential to ensure operational safety, support informed maintenance, and guide the development of targeted rehabilitation strategies.

Substantial research has been conducted on the degradation mechanisms and performance assessment of hollow-slab bridges. Field investigations and laboratory studies of retired or aging bridges have shown that many PC members retain substantial capacity even after decades of service. For example, Li et al.^2^ performed field testing of an existing hollow-slab bridge using trucks and proposed to strengthen the bridge using external prestressed tendons; Wang et al.^3^ conducted destructive tests to evaluate the degradation of mechanical performance of two PC hollow slabs which has been in service for 20 years; Yi et al.^4^ experimentally evaluated the shear performance of shallow hinge joints for prefabricated hollow slab bridges; Li and Song^5^ tested the mechanical performance of prestressed lightweight aggregate concrete hollow slab; and Liu et al.^6^ tested the residual flexural behavior of 28-year-old decommissioned PC hollow slabs. In the past several years, experimental research on strengthening or retrofitting techniques has been conducted. Various types of materials, such as carbon fiber-reinforced polymer (CFRP)^7,8^, self-consolidating cementitious composites^9^, ultra-high performance concrete^10^, and steel strips^11^, have been used to enhance the mechanical performance of hollow slabs. Beyond flexural performance and hinge joint behavior, the long-term dynamic characteristics of aged prestressed concrete members represent another critical aspect of serviceability. Bonopera et al.^12^ experimentally investigated the short-term vibration response of an uncracked prestressed concrete beam with a parabolic bonded tendon under long-age conditions (approximately 9.5 months). Their results demonstrated that the fundamental frequency of uncracked PC members is primarily sensitive to variations in the initial tangent Young’s modulus due to concrete consolidation or hardening, rather than to changes in the effective prestressing force or second-order initial curvature. This finding highlights that frequency-based methods may not be reliable indicators for prestress loss identification in uncracked PC girders, further underscoring the complexity of assessing the long-term performance of prestressed concrete structures.

Advances in analytical and computational methods have further enriched this understanding. Wang et al.^3^ constructed nonlinear finite element analysis (FEA) models, which were utilized to predict the mechanical behavior of two PC hollow slabs which has been in service for 20 years; Lai et al.^13^ performed FEA to analyze the mechanical behavior of PC hollow slabs and modified their deflection formula based on the Timoshenko theory; Chen et al.^14^ performed FEA to predict the mechanical performance of hollow-core slab bridges; Bi et al.^15^ conducted both theoretical and FEA studies on link slabs of hollow-slab bridges. Most recently, FEA has been performed to investigate the mechanical behaviors of reinforced truss hollow composite concrete slabs^16^, prestressed composite hollow slabs^17^, and concrete hollow slabs^18^. Collectively, these studies have advanced the knowledge of degradation mechanisms and assessment methods for reinforced concrete or bonded PC bridges. However, research on unbonded PC hollow slabs^19^ or beams^20^ remains sparse, although these structures are prone to aging-induced failures due to their unique prestressing configuration and modular assembly.

Previous studies on unbonded PC slabs or beams have largely concentrated on fundamental mechanical behavior, particularly the stresses of prestressed tendons and flexural capacity. For example, Cooke et al.^21^ conducted experimental testing of nine unbonded and three bonded PC slabs and compared the results with predictions using formulas; Harajli and Kanj^22^ studied the service load behavior of unbonded PC members; Au and Du^23^ predicted the ultimate stress in unbonded prestressed tendons. Zhang et al.^24^ studied the mechanical performance of curved slabs with unbonded tendons; Du et al.^25^ investigated the deflection of unbonded partially prestressed concrete continuous beams; and Moreira et al.^26^ developed a nonlinear FEA model for unbonded PC beams. In the past decade, research on unbonded PC structures has been extended to considering hazardous conditions, such as earthquakes^27^, fires^28,29^, gas explosion^30^, and low-velocity impacts^31^.

These studies offer critical insight into unbonded tendon mechanics and establish a sound theoretical framework for structural analysis. However, most of these studies focus on laboratory specimens with controlled materials and boundary conditions^32,33^. In practice, unbonded PC hollow-slab bridges undergo complex long-term deterioration, such as anchorage damage, hinge joint failure, and material degradation^34–36^, which alter load transfer and mechanical behavior. As a result, existing analytical models calibrated for pristine structures are insufficient to predict the residual behaviors of aged and deteriorated bridges under realistic service conditions.

Despite significant progress, three critical gaps have been identified from current knowledge: (1) Unbonded tendon-concrete interaction: The lack of bonding between tendons and concrete invalidates the plane-section assumption, making it difficult to model stress evolution in tendons under load, particularly after material aging. (2) Anchorage-dependent failure mechanisms: For aged hollow slabs, anchorage deterioration can lead to premature tendon rupture and brittle fracture before the section reaches flexural failure; yet this mechanism is seldom captured in existing evaluations. (3) Hinge-joint load redistribution: Hinge joints play an essential role in transferring loads among adjacent hollow slabs. Their degradation alters the load-sharing basis, increasing localized demand and accelerating bridge failure. However, limited studies have simultaneously considered hinge failure, anchorage degradation, and unbonded behavior. These limitations highlight the lack of integrated studies addressing the combined effects of structural aging, material degradation, and inter-slab interaction on the residual mechanical performance of unbonded PC hollow-slab bridges.

To address the above gaps, this study presents a comprehensive experimental investigation of the residual mechanical behavior of three full-scale unbonded PC hollow slabs extracted from a 20-year-old highway bridge exhibiting typical long-term deterioration, including anchorage zone cracking and hinge joint separation. The research framework integrates two complementary components: (i) field inspections to characterize existing damage patterns; (ii) destructive four-point bending tests to quantitatively evaluate residual cracking resistance, load-bearing capacity, failure modes, anchorage behavior, material properties, and degradation mechanisms. Unlike previous studies that relied on laboratory-conditioned or artificially aged specimens, this work leverages naturally aged, field-extracted components to capture the coupled effects of long-term service exposure on unbonded PC systems. Through full-scale experimental testing, this research aims to elucidate the predominant degradation mechanisms governing the long-term performance of unbonded PC hollow-slab bridges and to establish a mechanism-based evaluation framework to inform condition assessment and rehabilitation strategies for aging bridge inventories.

The remainder of the paper is organized as follows: “Bridge description and existing conditions” section elaborates the unbonded PC hollow-slab bridge and its existing conditions. “Experimental program” section introduces the experimental program including specimens and testing protocols. “Experimental results and discussion” section presents the results of experiments. “Conclusions” section summarizes the conclusions and recommendations.

## Bridge description and existing conditions

**Fig. 1:**
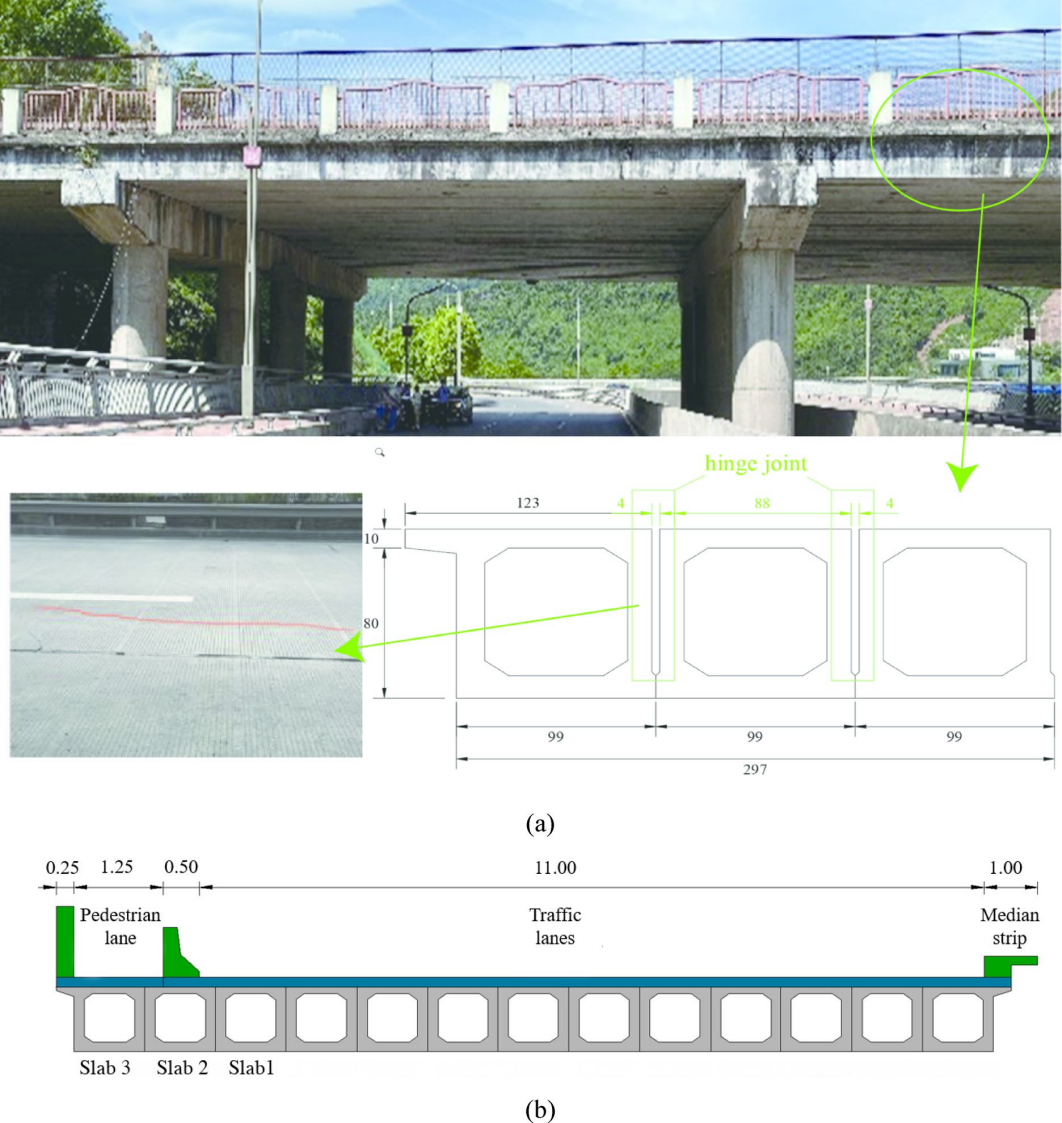

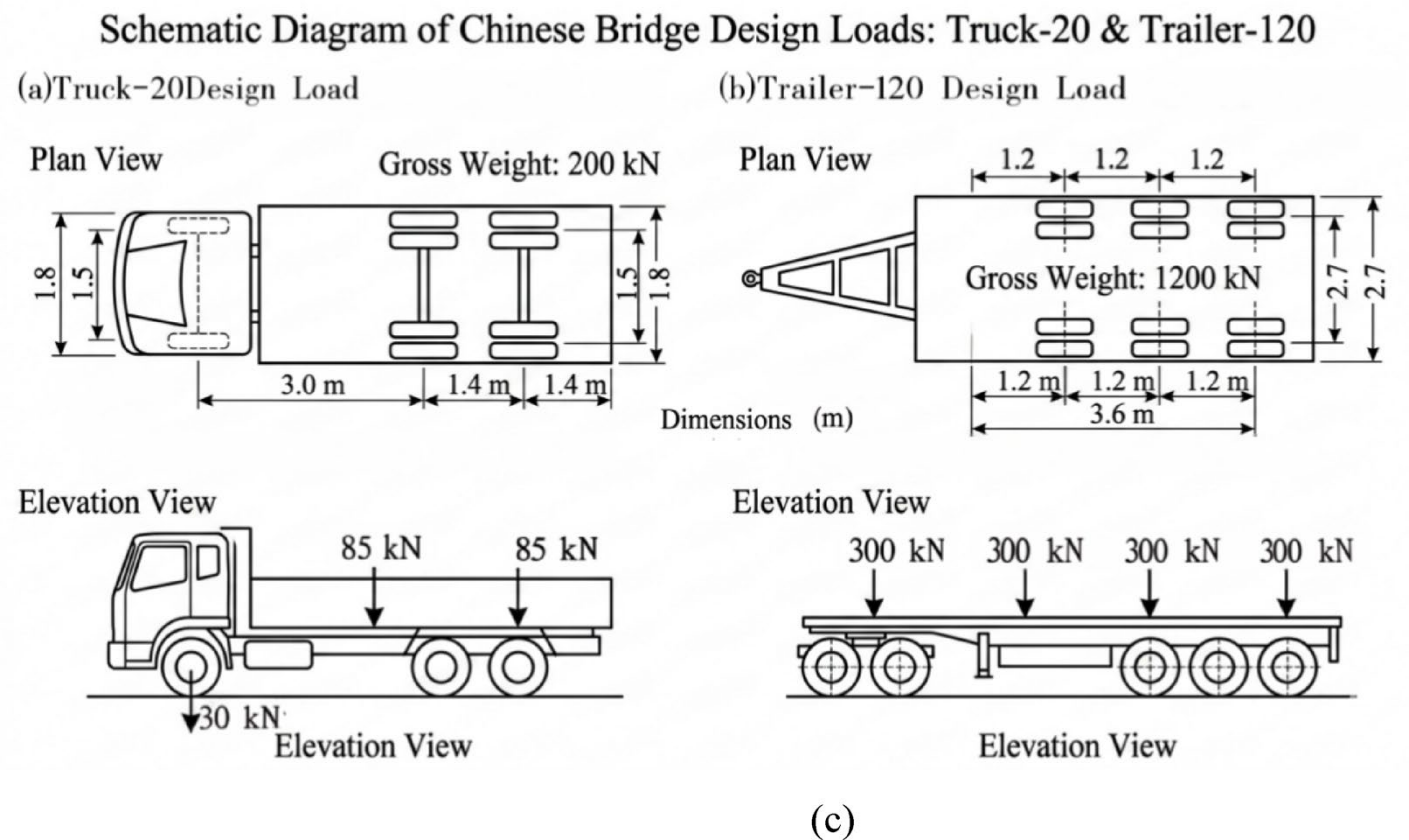
Studied bridge: (**a**) elevation view and detailed configuration; (**b**) transverse layout of the upstream side (unit: m); (**c**) schematic diagram of Chinese bridge design loads: Truck-20 and Trailer-120.

The investigated structure is a 20 m-long simply-supported PC hollow-slab bridge, as shown in Fig. 1a. The bridge deck is 27.5 m wide and consists of two separate carriageways (left and right). The left carriageway cross-section is shown in Fig. 1b. Each lane is 11 m wide, and the design load capacity accommodates 20-ton trucks and 120-ton trailers, which complies with the requirements of Chinese bridge design codes^37^. The axle spacings and axle loads of Truck-20 and Trailer-120 are shown in Fig. 1c. In addition, each side includes a 1.25 m-wide sidewalk designed for a pedestrian load of 3.5 kN/m^2^. The bridge deck slab, with a thickness of 13 cm, is made of steel-fiber-reinforced waterproof concrete, featuring a longitudinal gradient of 1.635% and a transverse slope of 2% for drainage. Beneath the bridge deck, 26 hollow slabs are arranged laterally. Each carriageway has 13 slabs, interconnected via hinge joints and supported at their ends by elastomeric bearing pads. The adjacent hollow slabs are interconnected by small keyway hinge joints, a typical configuration for bridges of this era. As illustrated in Fig. 1a, these joints feature a shallow keyway (approximately 40–50 mm in depth), thin mortar filling, and minimal transverse reinforcement with no tensile continuity across the joint. This configuration results in inherently weak transverse load transfer capacity. Each hollow slab is supported at its ends by elastomeric bearing pads. These bearings are designed to accommodate longitudinal movements caused by temperature variations and concrete shrinkage.

**Fig. 2:**
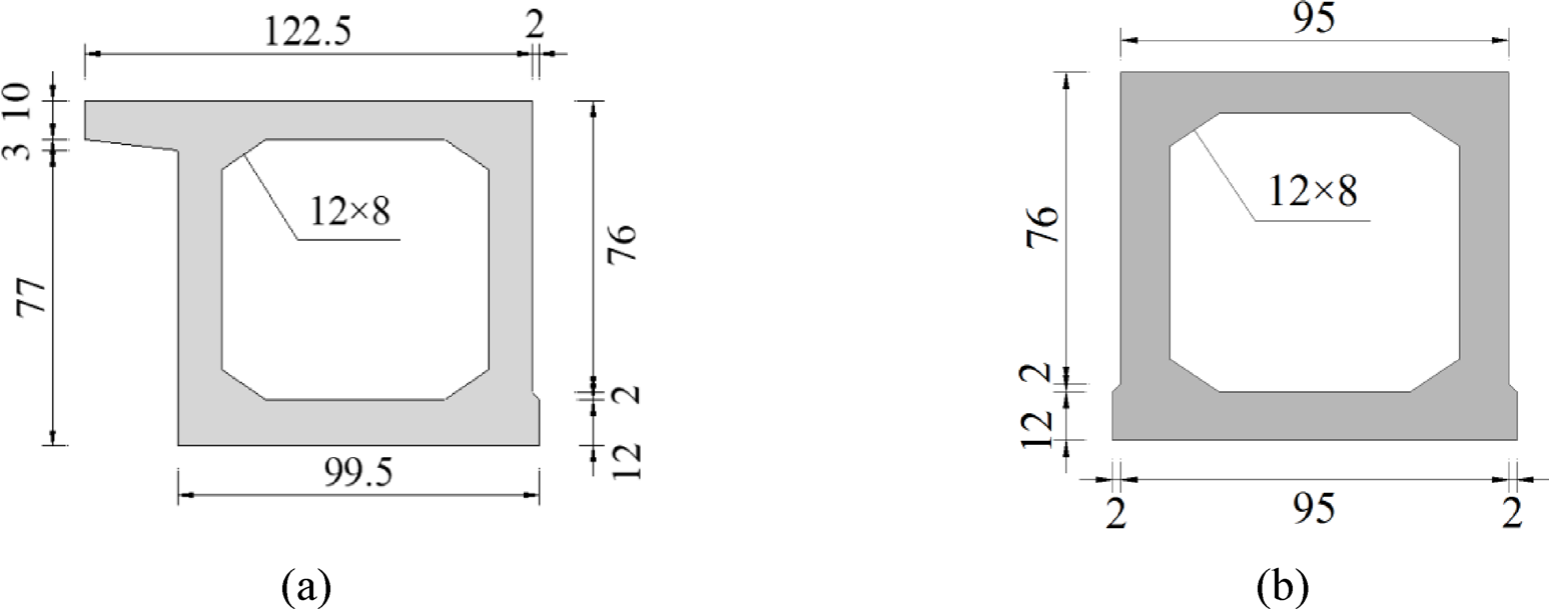
Hollow slabs: (**a**) exterior slab, and (**b**) interior slabs (unit: cm).

The cross sections of exterior and interior slabs are shown in Fig. 2. Each edge slab is 90 cm high, with a 10 cm-thick top flange and 12 cm-thick web and bottom flange. Chamfers of 12 cm × 8 cm are provided at the corners.

**Fig. 3:**
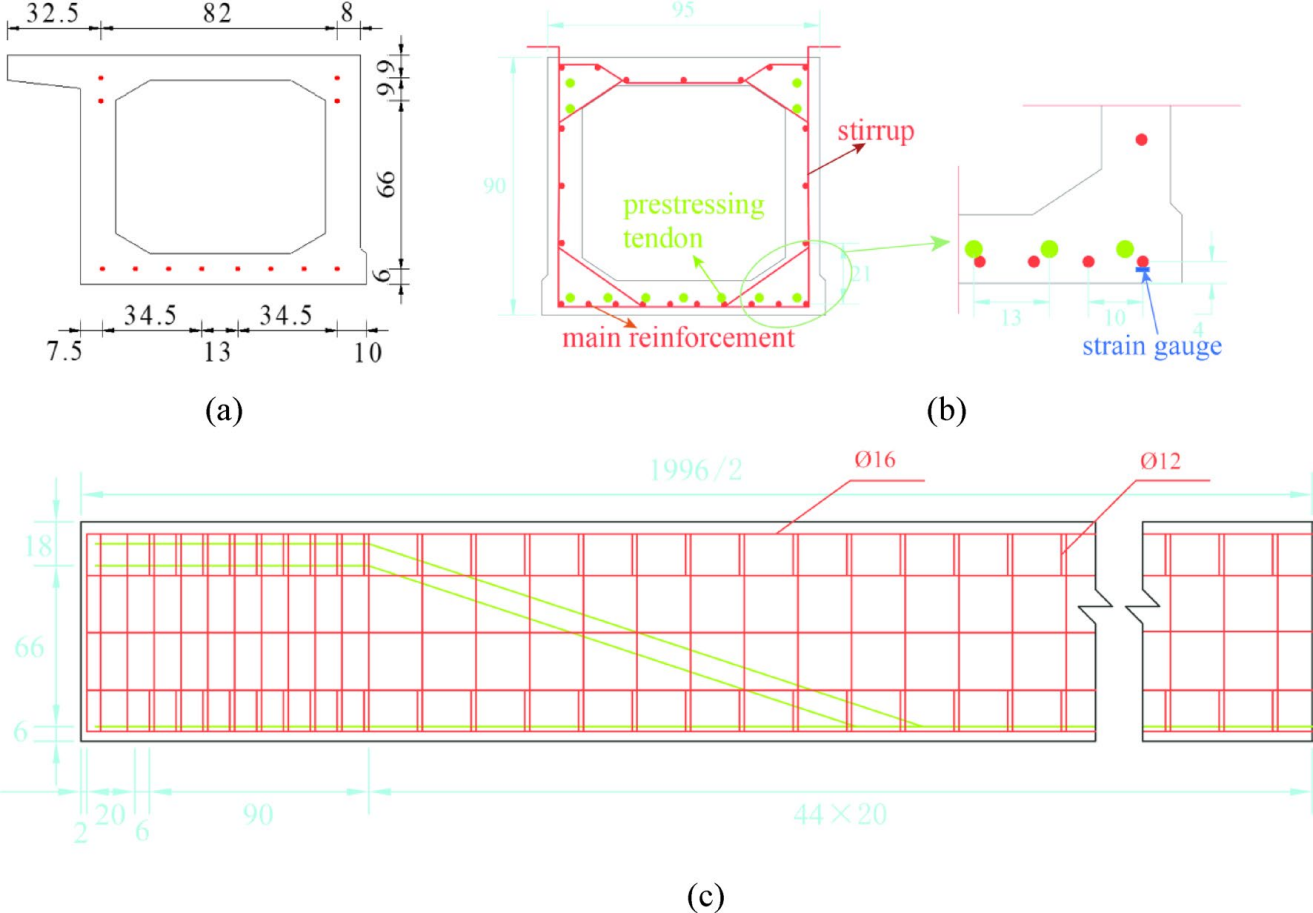
Layout of prestressed tendons (unit: cm): (**a**) cross section of an exterior slab, (**b**) Cross section of interior slab with reinforcement layout, and (**c**) Elevation layout of reinforcement and prestressing strands.

Each exterior slab contains 12 unbonded prestressed tendons, as shown in Fig. 3a. Eight tendons are straight and located at the bottom flange, while four curved tendons originate near the top of the webs at the ends and gradually transition to the bottom of the webs toward midspan. Each interior slab contains 11 tendons, as shown in Fig. 3b, seven straight tendons at the bottom flange and four curved tendons following a similar end-to-midspan transition, as shown in Fig. 3c. Each tendon consists of a low-relaxation high-strength steel strand, with a nominal diameter of 15.2 mm, a cross-sectional area of 140 mm^2^, a nominal tensile strength of 1860 MPa, and an initial jacking stress of 1395 MPa (75% of the nominal tensile strength) as specified in the original design. The tendons were passed through galvanized metal corrugated pipes which were embedded in the concrete. The corrugated pipes had an inner diameter of 70 mm and served as the ducts of the prestressed tendons.

**Fig. 4:**
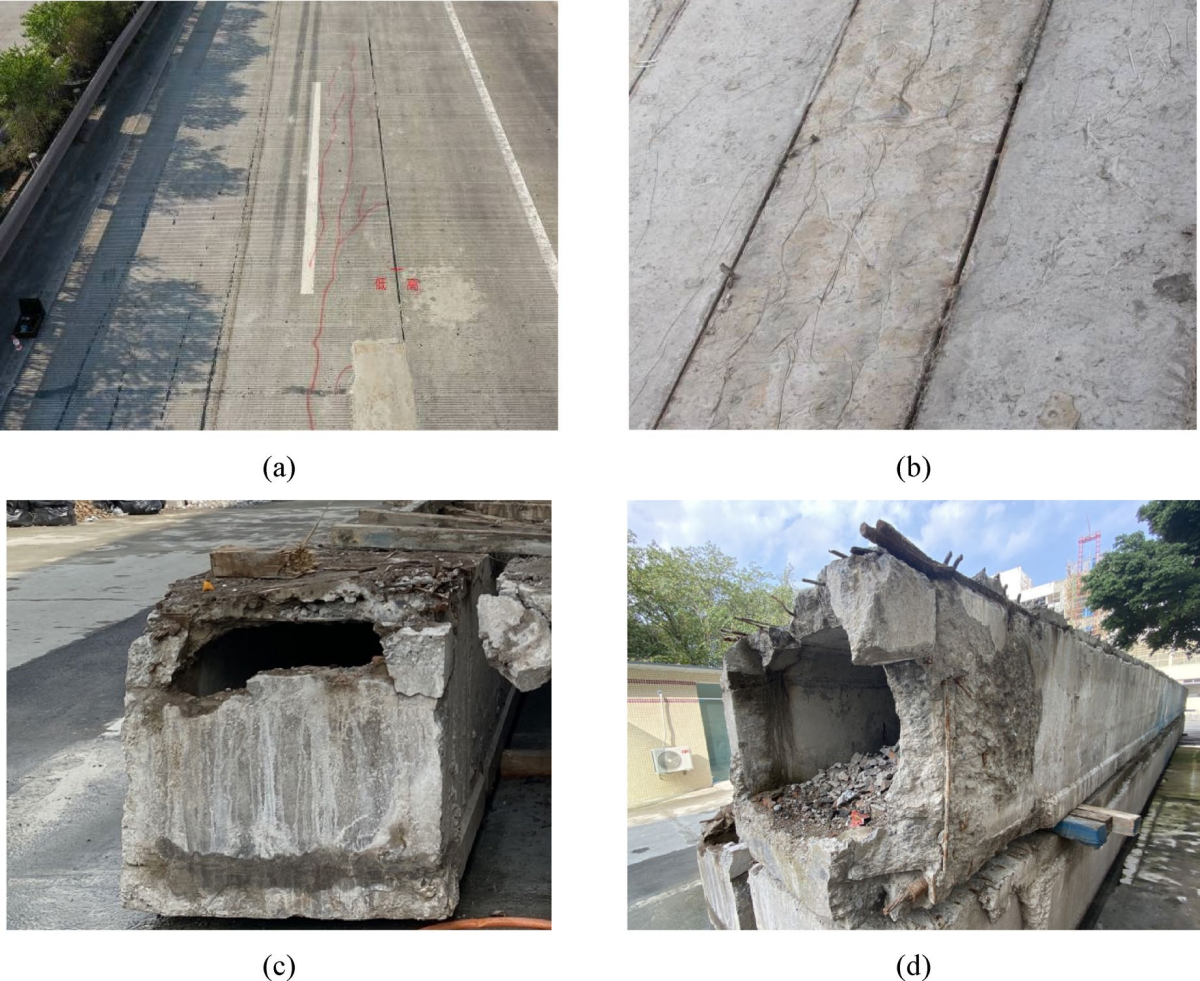
Representative damages: (**a**) longitudinal cracks in the deck, (**b**) hinge joint seperation, (**c**) anchorage damage of an interior slab, and (**d**) anchorage damage of an exterior slab.

After more than 20 years of service, the bridge has exhibited structural aging and multiple forms of deterioration, including cracking, deflection, hinge-joint failure, and concrete damage in the anchorage zones. The main defects are summarized as follows: (1) Longitudinal cracks along the hollow slabs: six locations identified, with a maximum crack length of 8.2 m and width of 0.12 mm. These cracks typically occur along the line of prestressing ducts and are attributed to the combined effects of concrete shrinkage and transverse tensile stresses induced by the prestressing force. (2) Longitudinal deck cracks: as shown in Fig. 4a, approximately 10 m in length and up to 1 mm in width. These are reflective cracks caused by underlying hinge joint separation, which disrupts the continuity of the deck pavement. (3) Hinge-joint detachment between slabs: as shown in Fig. 4b, resulting in weakened load-sharing among slabs and increased risk of overstressing in individual members. This detachment is primarily caused by accumulated shear fatigue and insufficient transverse reinforcement in the small keyway joints. (4) Anchorage zone damage: as shown in Fig. 4c, d, concrete cracking and spalling observed at prestressed tendon anchorages. This damage is attributed to long-term stress concentration and the absence of sufficient confining reinforcement in the end blocks*.* (5) Surface deterioration of slabs, including localized concrete spalling, honeycombing, and steel bar exposure. Given that these defects could largely affect structural safety and service life, three representative slabs were extracted from the bridge for experimental testing to evaluate their residual mechanical performance. Moreover, the observed hinge joint damage, characterized by visible separation and longitudinal cracking, provided the basis for defining the partial degradation scenario (50% stiffness reduction) in the subsequent finite element analysis.

## Experimental program

This section presents the experimental campaign of this research, including the specimens extracted from the bridge, flexural test setup, loading test protocols, as well as measurements and data acquisition during the experiments.

### Specimens and test setup

**Fig. 5:**
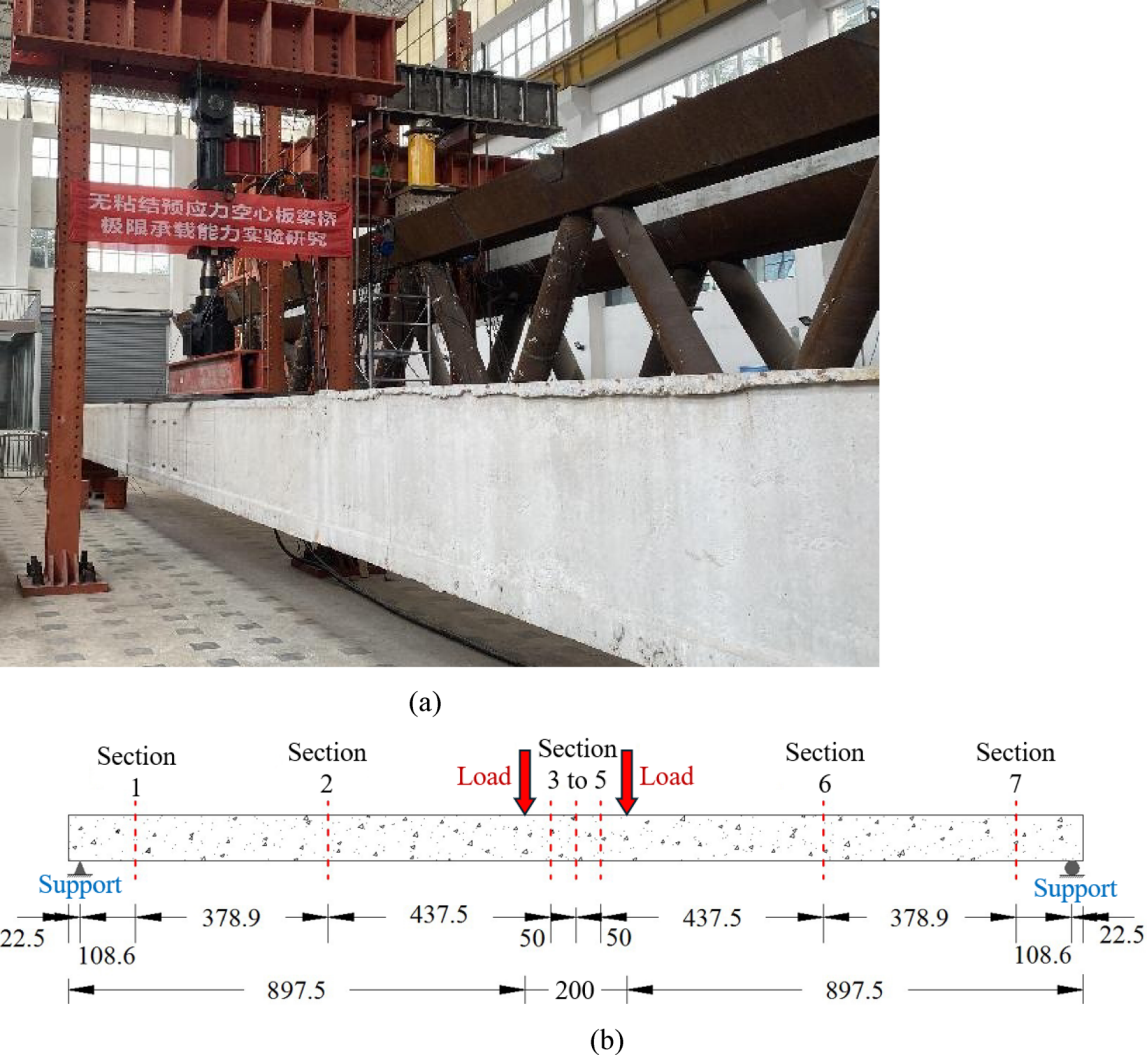
Experimental test setup: (**a**) photograph of Slab 1, and (**b**) layout of test setup (unit: cm).

Two interior slabs and one exterior slab were removed from the existing bridge for testing. They were designated as Slab 1, Slab 2, and Slab 3, respectively. All three slabs were subjected to four-point bending tests, as shown in Fig. 5. The three slab specimens were selected based on a comprehensive field inspection of all slabs in the target span (Zhouhe Bridge, left span 9). The selection aimed to capture a representative range of in-service damage conditions rather than choosing the least damaged elements. Slab 1 (interior) exhibited moderate anchorage damage, Slab 2 (interior) showed severe anchorage deterioration, and Slab 3 (exterior) was in relatively sound condition, providing a reference baseline. This sampling strategy ensures that the experimental results reflect the variability of actual bridge conditions after 20 years of service. Slabs with critical structural damage that could compromise handling safety were excluded.

The load was applied using a servo-controlled actuator with a load capacity of 1000 kN. A steel distribution beam was placed between the actuator and the slab to transfer the concentrated actuator force into two vertical loads, spaced 2 m apart, as shown in Fig. 5b.

### Test protocols

The experimental program consisted of five major loading and testing components, which were designed to comprehensively evaluate the mechanical behavior of the slab specimens:

1. Service load test

This test simulated the serviceability limit state of the bridge under the combination of dead load and live load^37^. Before loading, the design bending moment at the midspan section was calculated for this combination. During testing, the actuator load increased until the measured midspan moment matched the design moment. The load levels are summarized in Table 1.

(2) Ultimate load test

**Table 1:** Test loads of the hollow slabs under different conditions.

Designation of slabs	Category	Service load (kN)	Ultimate load (kN)
Slab 1	Interior	140	230
Slab 2	Interior	140	220
Slab 3	Exterior	170	250

This stage simulated the ultimate limit state of the bridge, and the ultimate load is expressed in Eq. (1) [36]. The theoretical midspan moment under this condition was calculated in advance, and the load was increased until this value. The applied loads are listed in Table 1.

1 $$L_{U} = 1.2D_{L} + 1.4L_{L}$$

where *D*_*L*_ denotes dead load, and *L*_*L*_ denotes live load.

(3) Failure load test

After completing the ultimate load test, the slabs were unloaded to zero. Subsequently, they were reloaded to failure using a hybrid control strategy. Force control was applied during the initial elastic stage and up to the onset of tensile reinforcement yielding, with a loading rate of approximately 0.2 kN/s. This allowed for accurate identification of cracking and yield loads. Following yielding, the loading protocol was switched to displacement control at a rate of 2 mm/min to capture the full post-yield behavior, including the softening branch and descending branch of the load–deflection curve, until structural failure occurred. This test aimed to investigate the actual load-bearing capacity, ductility, and failure mechanisms of the slabs.

(4) Residual anchorage performance test

Since the prestressed tendons were unbonded and connected to concrete only through end anchorages, the anchorage performance was critical to structural safety. After flexural failure of the three slabs, the anchorage devices, grips, and tendon bundles were tested to evaluate their residual anchorage strength and behavior.

(5) Material property tests

To assess potential material degradation due to long-term aging, core samples were extracted from the failed slabs. Tests were conducted to determine: (i) Concrete: compressive strength, tensile strength, and elastic modulus; (ii) Reinforcing steel: tensile strength, yield strength, elastic modulus, elongation after fracture, and elongation at maximum load; and (iii) Prestressed tendons: tensile strength, yield strength, elastic modulus, and elongation at maximum load.

### Measurements and data acquisition

During the tests, the following measurements were recorded: (1) Vertical displacements at key sections (Fig. 5): support section, midspan section, and “section 2” and “section 6” sections, measured using linear variable differential transformers (LVDTs) with an accuracy of 0.005 mm. (2) Concrete strains at the top, web, and bottom slabs in the midspan region, as well as the principal concrete strain near the end webs, measured using electrical-resistance strain gauges. (3) Strains of reinforcing bars in the bottom slab at midspan, measured using strain gauges bonded to the steel surface. (4) Cracking and failure loads of each slab, identified through visual observation and strain gauge monitoring. (5) Crack widths and propagation patterns during the loading process, recorded using a crack scope (precision: 0.02 mm) and marked on the slab surface at each load stage.

The strain gauges measured 80 mm in length. Their resistance, accuracy, and measurement ranges were 120 Ω, 1 με, and 5000 με, respectively. Cracks were measured using a crack scope (model: CS-150®, precision: 0.02 mm)^38^. Strains of reinforcing bars were measured using electrical-resistance strain gauges bonded to the surface of the main longitudinal tensile bars at the midspan sections. To access the bars, the 40 mm concrete cover was locally chiseled away, the bar surface was ground smooth and cleaned, and the gauges were bonded using cyanoacrylate adhesive. After installation, the gauges were protected and the chiseled area was repaired with epoxy mortar. The complete reinforcement layout and gauge locations are illustrated in Fig. 3b.

These sensors were connected to a data acquisition system (brand: National Instruments) for continuous measurements during the loading tests. The sampling frequency was set to 1 Hz for all sensors. To reduce the quantity of data in the postprocessing stage, the measurements were down-sampled to 0.1 Hz, consistent with reference^39^.

## Experimental results and discussion

Based on the experimental program in the last section, comprehensive experimental results were obtained. This section presents and discusses the results for evaluating serviceability, crack resistance, performance under ultimate loads, failure modes, load-carrying capacity, as well as anchorage conditions and residual material properties.

### Serviceability

**Fig. 6:**
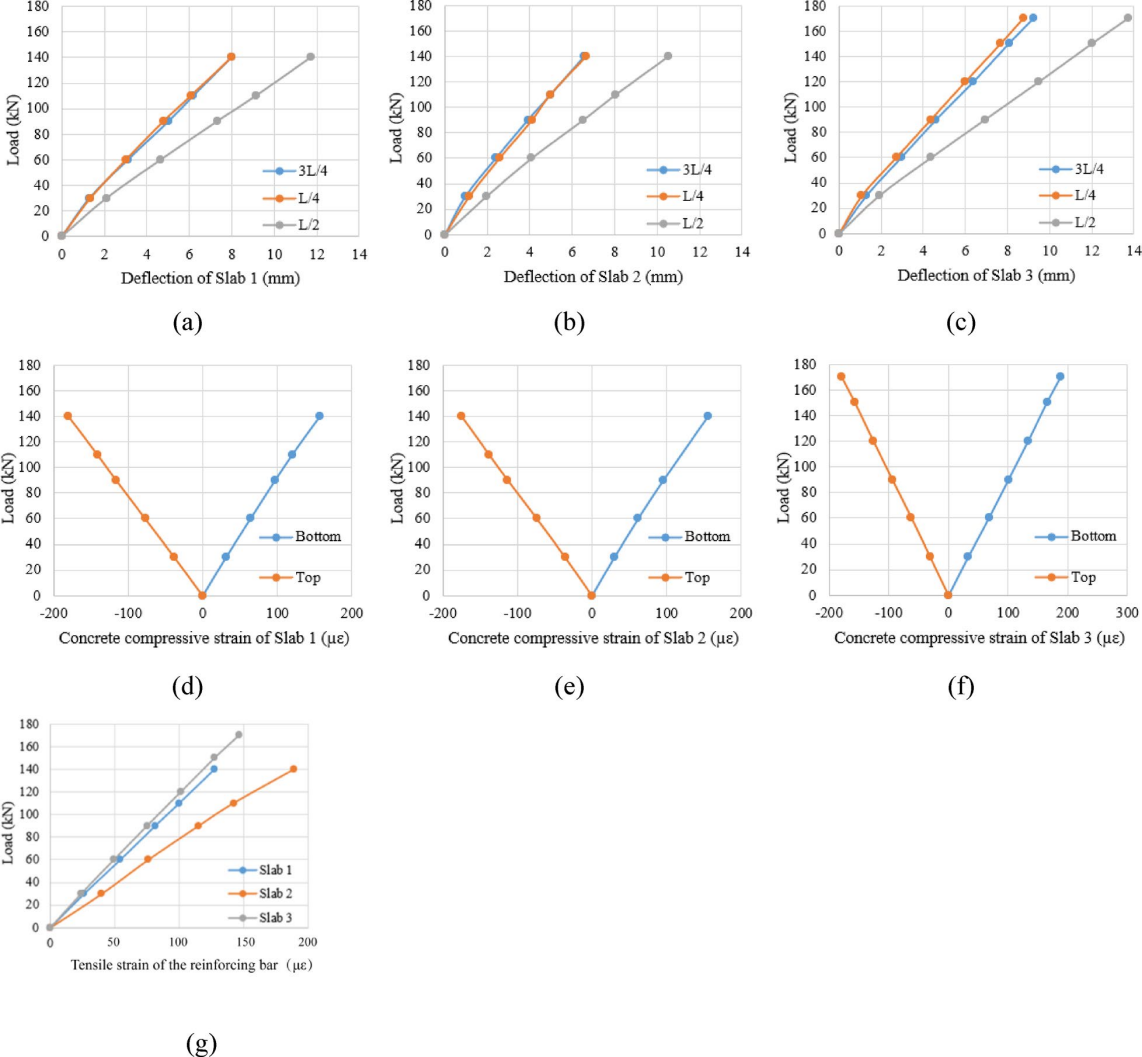
Test results of the slabs under service loads: (**a**) deflection of Slab 1, (**b**) deflection of Slab 2, (**c**) deflection of Slab 3, (**d**) concrete strain in Slab 1, (**e**) concrete strain in Slab 2, (**f**) concrete strain in Slab 3, and (**g**) bottom steel strain in the slabs.

Figure 6a–c show the load–deflection curves of the three slabs under design service loads. The deflections increase approximately linearly with load after the initial seating phase (beyond approximately 30 kN), indicating that the slabs were within the elastic range under design loads. The slight nonlinearity observed below 30 kN is attributed to initial settlement of the loading system, including compression of the elastomeric bearing pads and take-up of slack in the loading assembly—a common phenomenon in full-scale structural testing. The linear response from 30 kN upward confirms the elastic behavior of the slabs. The deflection verification coefficients ranged between 0.60–0.81 for Slab 1, 0.43–0.73 for Slab 2, and 0.55–0.94 for Slab 3, which are slightly outside the typical range of 0.7–1.0 specified in code JTG/T J21-01-2015^37^. It should be noted that the theoretical deflection values calculated according to the design codes were based on the actual measured material properties. According to the code, smaller coefficients may result from higher material strength, which is further examined in the following section. The residual deflection ratios were 1.25–1.87% (Slab 1), 1.14–1.67% (Slab 2), and 0–0.22% (Slab 3), all below the 20% limit defined in the code, confirming the elastic behavior of the three slabs.

As shown in Fig. 6d–f, concrete strain increased linearly with load, confirming the elastic stress–strain response of the slabs during the serviceability experiments. The concrete strain verification coefficients ranged from 0.65–0.87 (Slab 1), 0.64–0.84 (Slab 2), and 0.67–0.89 (Slab 3), aligning with the code’s allowable range (0.6–0.9). The midspan strain profiles exhibited linear variation along the section depth, verifying the plane-section assumption at the serviceability limit state. This demonstrates that the behavior of the specimens in the elastic phase is consistent with theoretical expectations, suggesting that the material properties are relatively well-maintained. Figure 6g shows that reinforcement stresses also increased linearly with load, remaining well within the elastic range. The bridge maintained satisfactory serviceability performance, meeting the design requirements. The theoretical midspan deflections under service loads were calculated according to the Chinese code JTG 3362–2018^40^ using the following equations:

2 $$K = \frac{P}{f} = \frac{\alpha \cdot B}{{L^{3} }}$$

3 $$B_{s} = 0.85E_{c} I_{0}$$

4 $$B = \frac{{M_{k} }}{{M_{q} \left( {\theta - 1} \right) + M_{k} }} \cdot B_{s}$$

where *P* is the concentrated load applied at midspan (N or kN), *f* is the corresponding midspan deflection (mm), *B* is the flexural stiffness of the member, *α* is a coefficient related to the loading form and boundary conditions (for a concentrated load at midspan, *α* = 48), Bs is the short-term stiffness, *Ec* is the elastic modulus of concrete, *I*_*0*_ is the moment of inertia of the transformed section (concrete and reinforcement/prestressing tendons converted to an equivalent concrete area based on the modular ratio), Mk is the bending moment under the standard load combination, *Mq* is the bending moment under the quasi-permanent load combination, and *θ* is the deflection magnification factor. For prestressed concrete flexural members, the code specifies *θ* = 2.0.

It should be noted that the theoretical calculations were performed using the actual measured material properties (concrete compressive strength of 61.8 MPa and elastic modulus of 37.8 GPa, as reported in “Anchorage conditions and residual material properties” section), rather than the original design values. Additionally, the calculated deflections were adjusted to exclude the effects of self-weight to enable direct comparison with the experimental measurements, which recorded only the load-induced deflections from the actuator.

The comparison between the theoretical and experimental midspan deflections under the design service load is presented in Table 2.

**Table 2:** Comparison of theoretical and experimental midspan deflections under design service load.

Slab	Experimental deflection (mm)	Theoretical deflection (mm)	Ratio (experimental/theoretical)
Slab 1	11.75	13.74	0.85
Slab 2	10.51	13.74	0.76
Slab 3	13.73	13.62	1.01

For Slabs 1 and 2 (interior slabs), the theoretical midspan deflections calculated according to the code-based method using actual material properties are 13.74 mm. However, when the contribution of the 13 cm thick concrete deck overlay is considered in the theoretical model, the predicted deflection reduces to 9.36 mm. The experimental deflections for Slabs 1 and 2 are 11.75 mm and 10.51 mm, respectively, yielding experimental-to-theoretical ratios of 0.85 and 0.76 relative to the code-based prediction (13.74 mm), and ratios of 1.26 and 1.12 relative to the deck-inclusive prediction (9.36 mm). The fact that the experimental deflections exceed the deck-inclusive theoretical predictions (ratios > 1.0) indicates that both interior slabs have experienced some loss of stiffness after 20 years of service, which is consistent with the observed anchorage deterioration and hinge joint damage documented in “Bridge description and existing conditions” section. In contrast, for Slab 3 (exterior slab located in the pedestrian area), which has no concrete deck overlay, the experimental deflection (13.73 mm) is in good agreement with the code-based theoretical value (13.62 mm), with a ratio of 1.01, confirming that the code-based prediction accurately reflects its actual behavior. Nevertheless, all three slabs satisfied the code requirements, confirming their adequate serviceability performance after 20 years of service.

### Cracking performance

Under design ultimate loads, cracks began to appear as the load increased. The results of crack loads and design service loads are listed in Table 3. For Slab 1, cracking occurred at 220 kN, with a maximum width of 0.04 mm and length of 0.6 m. For Slab 2, initial cracking occurred at 200 kN, and for Slab 3, at 190 kN, both with similar crack widths of 0.04 mm. Slab 3’s crack load was only 12% higher than the design service load, indicating limited cracking resistance.

Calculations using Eq. (5) from Chinese code GB 50204-2015^41^ showed that the crack resistance factor of Slab 3 was lower than the allowable limit (Table 4), confirming significant degradation in cracking performance.

5 $$\gamma_{cr} \ge \left[ {\gamma_{cr} } \right] = 0.95 \cdot \frac{{\sigma_{pc} + \gamma \cdot f_{tk} }}{{\sigma_{ck} }}$$

where *γ*_*cr*_ is the crack resistance factor; [*γ*_*cr*_] is the allowable crack resistance factor; *σ*_*pc*_ is the compressive stress of bottom concrete caused by the prestress of tendons;* γ* is the plasticity coefficient of the hollow-slab section; *f*_*tk*_ is the tensile strength of concrete; and *σ*_*ck*_ is the tensile stress of bottom concrete caused by the standard combined load.

**Table 3:** Results of cracking loads and design condition loads for the slabs.

	Slab 1	Slab 2	Slab 3
Crack load (kN)	220	200	190
Design service load (kN)	140	140	170
Load ratio	1.57	1.43	1.12

Since the tensile strength of concrete did not show notable deterioration, the reduced cracking resistance is likely caused by prestress losses accumulated over time. The magnitude and causes of prestress loss require further investigation. Moreover, partial hinge-joint failure between slabs may increase the load demand on individual slabs during service, raising cracking risks. A quantitative evaluation of this scenario is presented in the earlier section.

### Ultimate state results

**Fig. 7:**
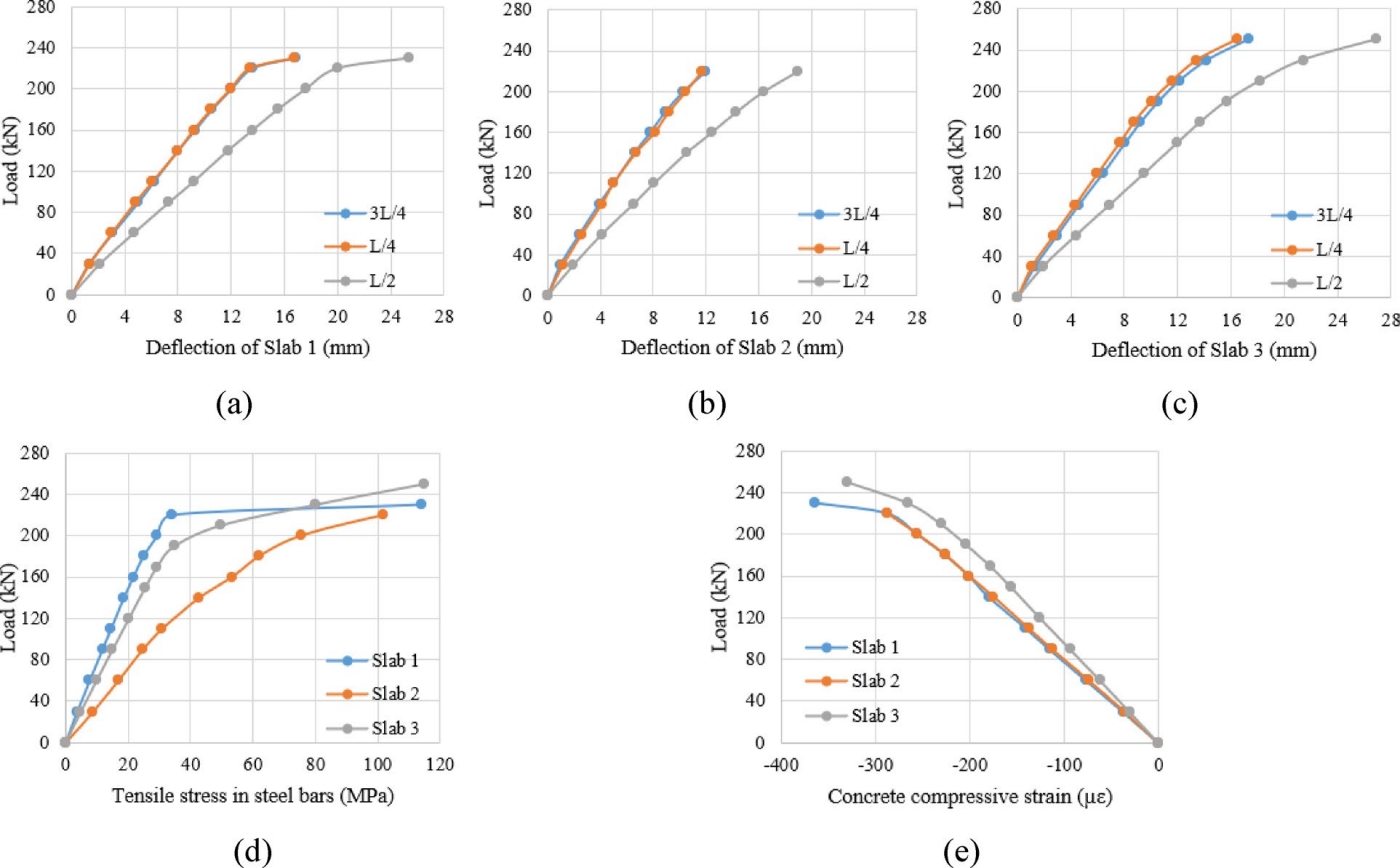
Test results of the slabs under design ultimate state loads: (**a**) deflection of Slab 1, (**b**) deflection of Slab 2, (**c**) deflection of Slab 3, (**d**) stresses of bottom steel bars, and (**e**) concrete strain at the top of the slabs.

The load–deflection curves under design ultimate state loads are shown in Fig. 7a–c. Before cracking, deflection increased linearly; after cracking, nonlinear behavior emerged. None of the three slabs failed under the ultimate state load, indicating that design-level strength requirements were met.

Figure 7d shows the stress-load curves of steel bars. Before cracking, the stresses of steel bars increased linearly. After cracking, the stresses increased more rapidly as concrete in the tension zone progressively ceased to be effective. At the design ultimate load, the measured steel stresses were 114 MPa (Slab 1), 102 MPa (Slab 2), and 115 MPa (Slab 3), all well below the yield strength of 404.5 MPa, indicating adequate safety margins. Notably, the steel stresses in Slab 2 are consistently lower than those in Slab 1 under equivalent loads. This difference is attributed to Slab 2’s less developed cracking pattern at the design ultimate load, which resulted in greater tension stiffening—more tensile stress being carried by the concrete between cracks, thereby reducing the stress demand on the reinforcing steel. In contrast, Slab 1 exhibited more extensive cracking, leading to higher steel stresses. Slab 3, with its larger cross-section, inherently experienced lower steel stresses despite its lower cracking load. Similarly, the compressive strain at the top fibers of concrete remained below − 364 με, as shown in Fig. 7e, far less than the ultimate compressive strain of concrete. The magnitudes of strains in Slab 3 are lower than those in Slab 1 and Slab 2 under the same load because Slab 3 has larger cross sections than the other two slabs (Fig. 2). These results indicate that the bridge retained sufficient load-bearing capacity after more than two decades of service.

### Failure modes and load-carrying capacity

**Fig. 8:**
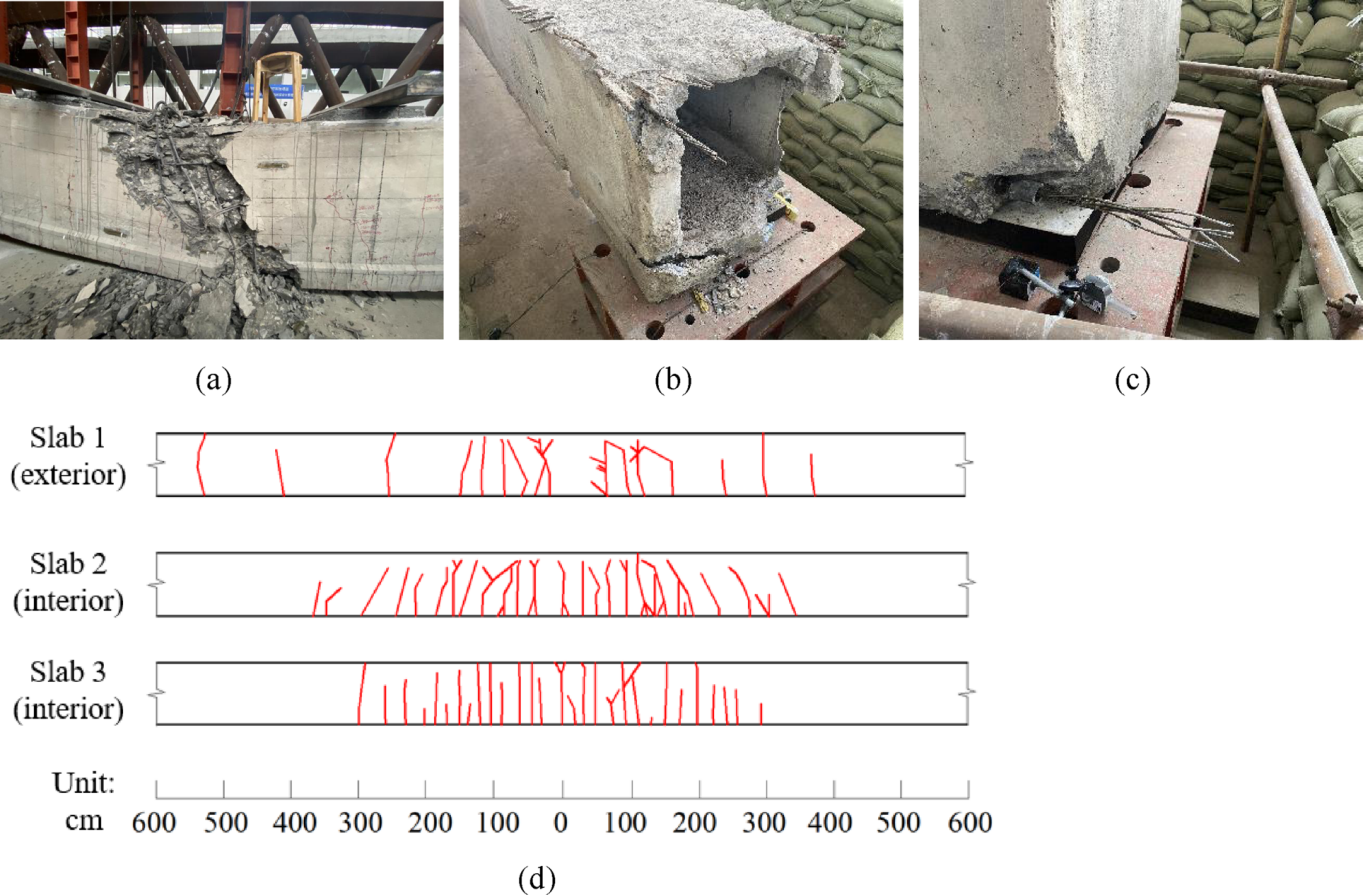
Failure modes of the hollow slabs: (**a**) Slab 1, (**b**) Slab 2, (**c**) Slab 3, and (**d**) crack pattern.

The three slabs exhibited two distinct failure modes (Fig. 8): (1) concrete crushing (Slab 1), associated with higher deflection and higher deformability; and (2) tendon fracture (Slabs 2 and 3), characterized by brittle failure and limited deformability. Crack patterns aligned with general unbonded PC behavior—fewer, widely spaced cracks.

**Fig. 9:**
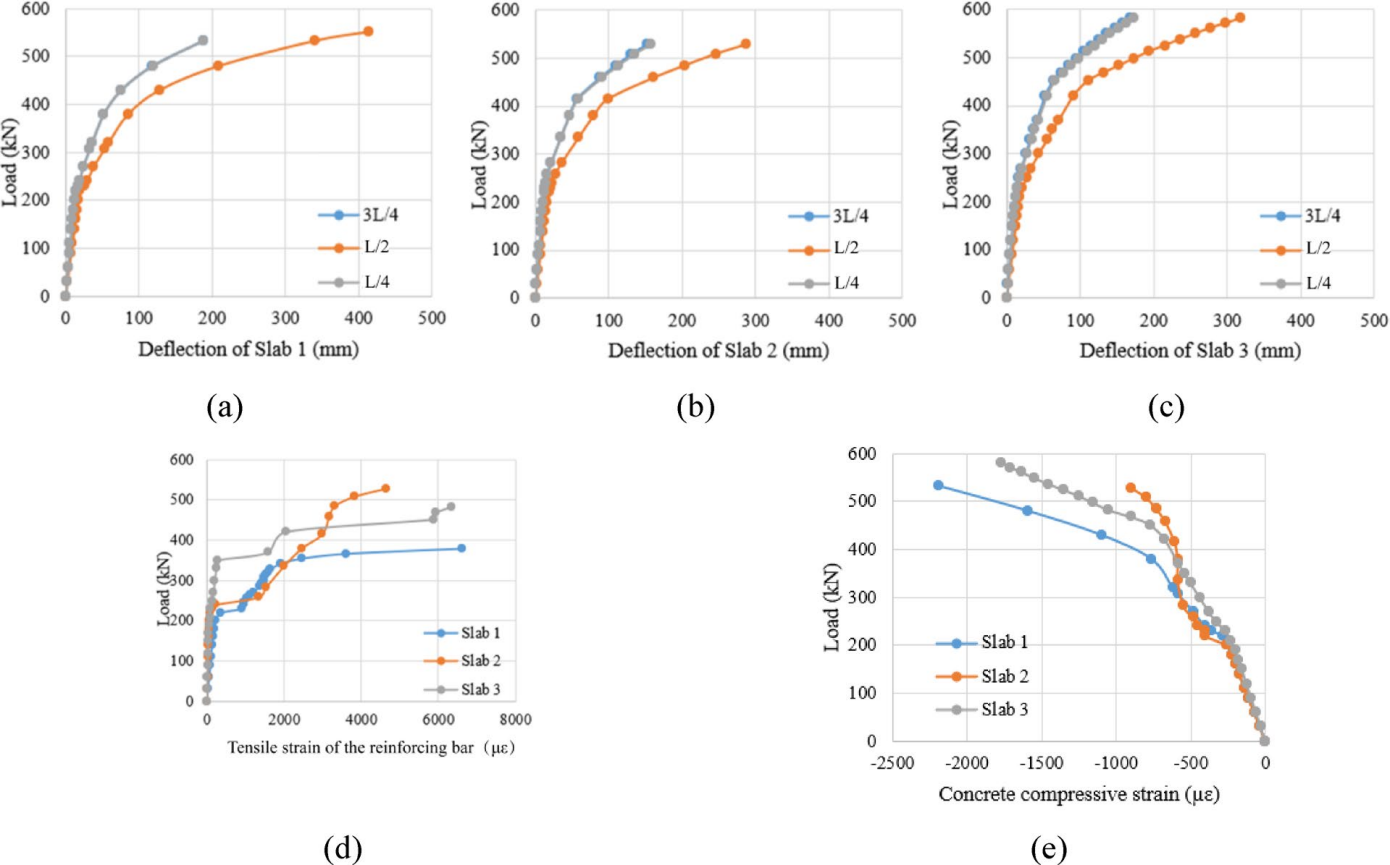
Test results of the slabs under failure loads: (**a**) deflection of Slab 1, (**b**) deflection of Slab 2, (**c**) deflection of Slab 3, (**d**) stresses of bottom steel bars, and (**e**) top concrete strains.

Under failure loads, all slabs exhibited nonlinear behavior (Fig. 9). Slab 1 failed at 551 kN with concrete crushing at the top slab. The maximum deflection reached 413.6 mm, showing ductile failure. Slab 2 failed at 529 kN when prestressed tendons ruptured at the anchorage, with a maximum deflection of 287.7 mm. Slab 3 failed at 581 kN due to tendon rupture near the end anchorage, with a deflection of 319.0 mm.

Reinforcement yielded at 307 kN (Slab 1), 297 kN (Slab 2), and 330 kN (Slab 3). The corresponding ductility factors (Table 5), calculated according to Eq. (6)^42^, indicate that the slabs retained significant nonlinear deformation capacity after long-term aging. However, the ductility coefficients for Slabs 2 and 3 decreased by 3.84 and 2.88, respectively, compared to Slab 1, reflecting the detrimental impact of anchorage-end fracture of prestressed tendons. Compressive strain development at the top fibers of concrete also revealed distinct trends. In Slab 1, strain reached − 2191 με before crushing, while Slabs 2 and 3 exhibited lower peak strains (− 897 με and − 1769 με, respectively), consistent with premature brittle failures.

6 $$\mu = \frac{\Delta u}{{\Delta y}}$$

where *μ* is the deflection ductility coefficient; △*u* is the failure deflection or the deflection when the slab fails; and △*y* is the yielding deflection or the deflection when yielding occurs.

**Table 4:** Analysis results of crack resistance for the hollow slabs.

	γ_cr_	[γ_cr_]
Slab 1	1.23	1.21
Slab 2	1.31	1.21
Slab 3	1.07	1.17

Safety coefficients were calculated according to Eqs. (7) and (8), using the design, ultimate, and failure loads (Table 6). The results show that the bridge’s design incorporated considerable safety margins, ensuring adequate residual strength despite long-term degradation.

7 $$\delta_{d} = \frac{{F_{u} - F_{d} }}{{F_{d} }}$$

8 $$\delta_{n} = \frac{{F_{u} - F_{n} }}{{F_{n} }}$$

where *δ*_*d*_ is the operational safety coefficient; *δ*_*n*_ is the ultimate safety coefficient; *F*_*u*_ is the failure load; *F*_*d*_ is the design operational load; and *F*_*n*_ is the design ultimate load.

**Table 5:** Results of critical loads and deflections.

	Yielding load (kN)	Yielding deflection (mm)	Failure load (kN)	Failure deflection (mm)	Ductility factor
Slab 1	307	32.8	551	413.6	12.6
Slab 2	297	42.4	529	287.7	8.8
Slab 3	330	55.3	581	319.0	9.7

### Anchorage conditions and residual material properties

**Fig. 10:**
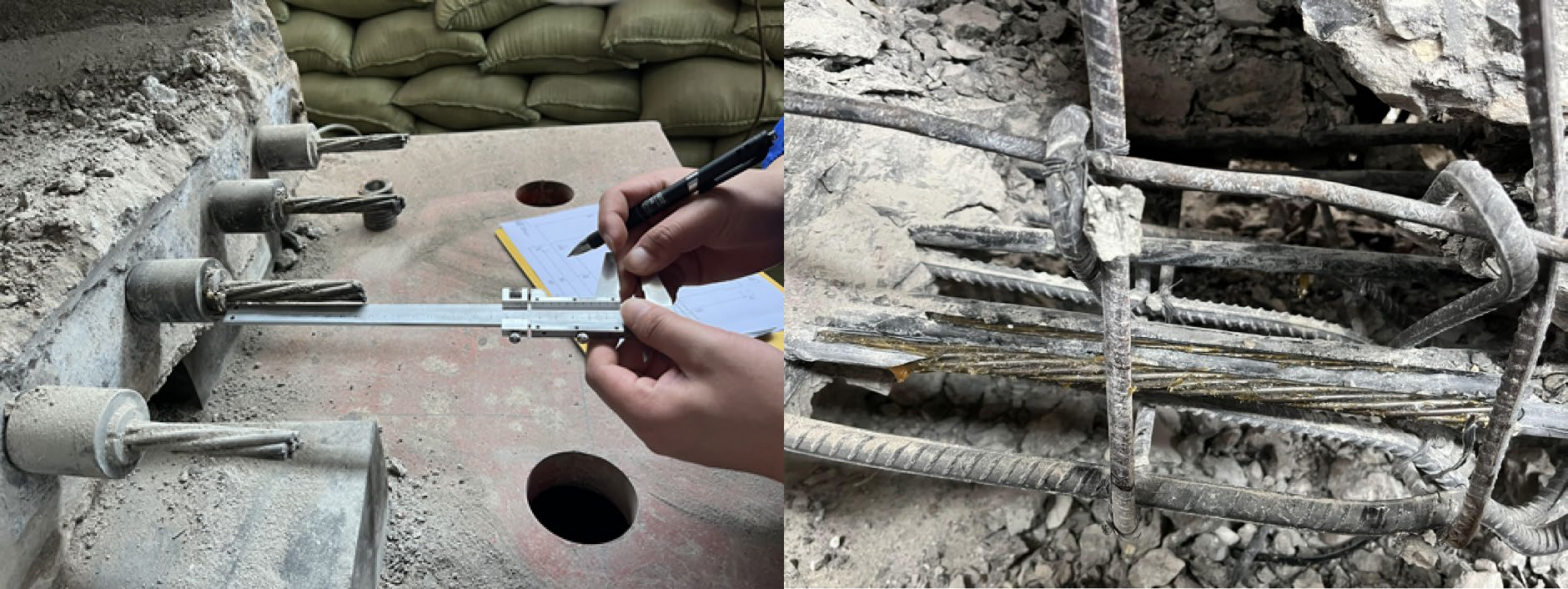
Prestressing strands: (**a**) end anchorage, (**b**) midspan region.

As shown in Fig. 10, a visual inspection of the anchorage zone revealed no significant corrosion or damage to the prestressing tendons, anchorages, or wedges. Following the flexural failure tests, the three slabs were cut open to allow for direct observation of the condition of the unbonded tendons along their entire length. Despite 20 years of service, the tendons—encased in plastic sheathing and filled with anti-corrosion grease—exhibited no visible signs of corrosion, thereby confirming the effectiveness of the original corrosion protection system. However, subsequent anchorage tests showed that the total elongation at the maximum load was 1.0–1.4%, below the minimum 2% required by codes JGJ 85-2010^43^ and JGJ 92-2004^44^. The anchorage efficiency factors were in the range of 0.84 to 0.88, lower than the 0.95 requirement, indicating significant degradation in anchorage zones. The deficiency poses potential safety risks for continued service.

The reduced anchorage efficiency (0.84–0.88) and limited tendon elongation (1.0–1.4%) observed in Slabs 2 and 3 are attributed to a combination of factors rather than a single cause. All fractures occurred at the wedge-gripping zone, where strands are subjected to high local stress concentrations. This inherent stress concentration, combined with long-term fatigue under service loads, likely reduced strand ductility over 20 years. Post-failure inspections revealed localized rust only where protective sheathing was damaged by concrete cracking (Fig. 4b, c), indicating that generalized corrosion was not the primary mechanism. Notably, strand base material retained adequate tensile strength (1961 MPa), confirming that embrittlement was localized to anchorage zones rather than occurring along the span. The correlation between pre-test damage ratings and failure loads—Slab 2 (severely damaged) failed at 529 kN, while Slab 3 (relatively sound) reached 581 kN—further supports that cumulative long-term degradation, not a single defect, governed anchorage performance. These findings highlight that anchorage zones are the critical vulnerability in unbonded PC systems.

After the testing of the three slabs, samples were taken from undamaged web regions to evaluate the residual material properties of concrete, steel bars, and prestressed tendons (Table 7). Reference allowable values were obtained from codes (JTJ 023-85^45^, GB 1499.2-2007^46^, GB/T 5224-2003^47^, and JGJ 92-2004^43^). It should be noted that the design value listed in the table corresponds to the historical design specification used at the time of bridge construction, whereas the measured value represents the average compressive strength obtained from laboratory tests of extracted concrete samples. Therefore, these two values have different statistical meanings and are not intended for direct comparison. The relatively high measured compressive strength does not necessarily indicate the use of modern high-strength concrete. Because the bridge was constructed more than two decades ago, the quality control during on-site construction was less standardized, and the actual concrete strength could exceed the nominal design grade. In addition, long-term hydration and aging effects may further increase the measured compressive strength of the concrete. Except for the slightly reduced elastic modulus of reinforcing steel, all measured parameters exceeded the historical design limits, indicating that the materials retained satisfactory mechanical strength and stiffness after two decades of service. The compressive strength of concrete was higher than its design value, supporting the earlier inference in the previous section that high material strength contributed to smaller deflection coefficients.

**Table 6:** Operating safety reserve coefficient and safety coefficients.

	Design operational loads (kN)	Design ultimate loads (kN)	Failure loads (kN)	Operational safety coefficients	Ultimate safety coefficients
Slab 1	140	230	551	2.93	1.39
Slab 2	140	220	529	2.78	1.40
Slab 3	170	250	581	2.42	1.32

**Table 7:** Test results of the material performances.

	Concrete	Limit	Steel bars	Limit	Tendons	Limit
Compressive strength (MPa)	61.8	28.0	–	–	–	
Tensile strength (MPa)	5.7	2.6	595	455	1961	1860
Elastic modulus (GPa)	37.8	33.0	188	200	203	195
Yielding strength (MPa)	–	–	404.5	335.0	1769	1671
Elongation at failure (%)	–	–	30	17	–	
Elongation at maximum load (%)	–	–	14.6	7.5	6.4	3.5

## Conclusions

This paper has presented an experimental investigation into the residual mechanical behavior of a deteriorated unbonded prestressed concrete (PC) hollow-slab bridge after more than 20 years of service. The primary objectives were to quantify the residual cracking resistance, load-bearing capacity, and failure mechanisms of naturally aged slabs through full-scale destructive testing, and to evaluate the effects of anchorage degradation on structural performance. Based on the comprehensive experimental investigations, the following major conclusions are drawn:

1. After over two decades of service, the bridge retained sufficient load-carrying capacity to satisfy current design code requirements. The concrete, reinforcement, and prestressed tendons exhibited mechanical strengths substantially exceeding their original design values, with concrete compressive strength reaching 61.8 MPa, confirming that material aging alone was not the primary cause of performance degradation.
2. The exterior slab demonstrated critically reduced cracking resistance, with a cracking load of only 190 kN—merely 12% above the design service load. This finding highlights the essential role of transverse load distribution in preventing premature cracking in individual slabs, and underscores the need to maintain hinge joint integrity to ensure effective load sharing among adjacent slabs.
3. Despite satisfactory material properties, the end anchorage systems exhibited significant degradation. Anchorage efficiency coefficients ranged from 0.84 to 0.88, below the code requirement of 0.95, and tendon elongation at maximum load was only 1.0–1.4%, far below the 2% minimum standard. Consequently, two slabs experienced premature brittle failure due to tendon rupture at the anchorage, with ductility coefficients reduced by up to 30% compared to the ductile failure observed in Slab 1. The residual flexural capacity decreased by 3.2–5.2% relative to theoretical values, primarily attributable to anchorage deterioration rather than material aging. This quantitatively establishes anchorage degradation as the governing mechanism for capacity loss in aged unbonded PC structures.

The findings demonstrate that conventional condition assessments focusing solely on material properties may underestimate structural risk, as critical degradation can be concentrated in anchorage zones and hinge joints. Accordingly, maintenance strategies should prioritize routine inspection and monitoring of anchorage systems using non-destructive techniques, rehabilitation of damaged hinge joints to restore transverse load transfer, and development of targeted strengthening solutions for anchorage zones in vulnerable slabs.

While this study has elucidated the dominant role of anchorage and hinge joint degradation through full-scale destructive testing, further research is needed to investigate the interactive mechanism between prestressing strands and the main girder under long-term service conditions. Specifically, the load transfer behavior at the strand–concrete interface, the evolution of stress distribution in unbonded systems with anchorage deterioration, and the development of predictive models for strand stress redistribution warrant systematic investigation. Additionally, long-term monitoring of in-service bridges using distributed fiber optic sensing could provide valuable data to validate and refine these models, ultimately contributing to more reliable life-cycle performance assessments and extended service life for aging unbonded PC hollow-slab bridges.

## Data Availability

Correspondence and requests for materials should be addressed to Xiaobin Li.

## List of symbols

*A*_*p*_: Area of prestressing tendon (mm^2^)
*A*_*s*_: Area of reinforcing steel bar (mm^2^)
$A_{s}^{\prime }$: Area of compression reinforcement (mm^2^)
$b_{f}^{\prime }$: Width of compression flange (mm)
*B*: Flexural stiffness of member (N mm^2^)
*B*_*s*_: Short-term stiffness (N·mm^2^)
*D*_*L*_: Dead load (kN)
*E*_*c*_: Elastic modulus of concrete (MPa)
*E*_*s*_: Elastic modulus of reinforcing steel (MPa)
f: Deflection (mm)
f_cd_: Design compressive strength of concrete (MPa)
f_pd_: Design tensile strength of prestressing tendon (MPa)
f^sd^: Design tensile strength of reinforcing steel (MPa)
f_td_: Design tensile strength of concrete (MPa)
f_tk_: Tensile strength of concrete (MPa)
*F*_*d*_: Design operational load (kN)
*F*_*n*_: Design ultimate load (kN)
*F*_*u*_: Failure load (kN)
h: Section height (mm)
$h_{f}^{\prime }$: Thickness of compression flange (mm)
*I₀*: Moment of inertia of transformed section (mm^4^)
*K*: Stiffness coefficient (N/mm)
*L*: Span length (mm)
*L*_*L*_: Live load (kN)
*L*_*U*_: Ultimate load (kN)
*Mₖ*: Bending moment under standard load combination (kN m)
*M*_*q*_: Bending moment under quasi-permanent load combination (kN m)
*P*: Concentrated load at midspan (kN)
x: Compression zone height (mm)
*α*: Coefficient related to loading form and boundary conditions (–)
*γ*: Plasticity coefficient of hollow-slab section (–)
*γ*_*cr*_: Crack resistance factor (–)
[*γ*_*cr*_]: Allowable crack resistance factor (–)
*δ*_*d*_: Operational safety coefficient (–)
*δ*_*n*_: Ultimate safety coefficient (–)
*Δ*_*u*_: Failure deflection (mm)
*Δ*_*y*_: Yielding deflection (mm)
*ε*: Strain (με)
*θ*: Deflection magnification factor (–)
*μ*: Deflection ductility coefficient (–)
*σ*_*ck*_: Tensile stress of bottom concrete under standard load (MPa)
*σ*_*pc*_: Compressive stress of bottom concrete due to prestress (MPa)
